# Generation and evaluation of input values for computational analysis of transport processes within tissue cultures

**DOI:** 10.1002/elsc.202100128

**Published:** 2022-02-23

**Authors:** Ehsan Fattahi, Shahed Taheri, Arndt F. Schilling, Thomas Becker, Ralf Pörtner

**Affiliations:** ^1^ Institute of Bioprocess and Biosystems Engineering Hamburg University of Technology Hamburg Germany; ^2^ Chair of Brewing and Beverage Technology TUM School of Life Sciences Technische Universität München Freising Germany; ^3^ Department of Trauma Surgery Orthopaedics and Plastic Surgery University Medical Center Göttingen Göttingen Germany

**Keywords:** computational models, experimental techniques, fluidics, organotypic tissue cultures, transport processes

## Abstract

Techniques for tissue culture have seen significant advances during the last decades and novel 3D cell culture systems have become available. To control their high complexity, experimental techniques and their Digital Twins (modelling and computational tools) are combined to link different variables to process conditions and critical process parameters. This allows a rapid evaluation of the expected product quality. However, the use of mathematical simulation and Digital Twins is critically dependent on the precise description of the problem and correct input parameters. Errors here can lead to dramatically wrong conclusions. The intention of this review is to provide an overview of the state‐of‐the‐art and remaining challenges with respect to generating input values for computational analysis of mass and momentum transport processes within tissue cultures. It gives an overview on relevant aspects of transport processes in tissue cultures as well as modelling and computational tools to tackle these problems. Further focus is on techniques used for the determination of cell‐specific parameters and characterization of culture systems, including sensors for on‐line determination of relevant parameters. In conclusion, tissue culture techniques are well‐established, and modelling tools are technically mature. New sensor technologies are on the way, especially for organ chips. The greatest remaining challenge seems to be the proper addressing and handling of input parameters required for mathematical models. Following Good Modelling Practice approaches when setting up and validating computational models is, therefore, essential to get to better estimations of the interesting complex processes inside organotypic tissue cultures in the future.

**ABBREVIATIONS**
CHO: chinese hamster ovary cells
CPPs: critical process parameters
CQA: critical quality attributes
DTs: digital twins
ECM: extracellular matrix
MOC: multi‐organ‐on‐a‐chip
OC: organ‐on‐a‐chip
OTR: oxygen transfer rate
PAT: process analytical technologies

## 1 INTRODUCTION

‘Organotypic tissue culture’ refers to a micro‐structured cell culture under in vitro conditions that consider the three‐dimensionality (3D) and the physiology of the tissue. The ultimate goal is to create a vital tissue with organ‐specific structure and functionality, mimicking the complexity of the cellular microenvironment in vivo. Areas of application mainly cover basic research (e.g., stem cell research, tumour models), pharmacology and toxicity tests, regenerative medicine (tissue engineering for implantation), and in vitro meat, among others [1, 2]. Techniques for tissue cultivation have seen significant advances during the last decades and novel 3D cell culture systems have become available, for example, 3D organoids or organ/body‐on‐chip systems [3, 4, 5, 6].

A tissue culture construct is mainly composed of cells, biomaterials/carrier systems and solutes, which are cultivated in a bioreactor. Bioreactors allow to control micro‐environmental parameters and recapitulate tissue‐specific cell–cell contacts or cell–matrix interactions, mimic gradients with respect to oxygen, nutrients, waste products or pH, diffusion of regulatory molecules, internal and external mass transfer (e.g., oxygen, nutrient, and waste materials transfer), as well as different kinds of stimulation (mechano‐, electro‐, thermos‐ and magneto‐transduction) [1, 7]. The function of the cells in a tissue culture is strongly governed by fluidics and transport processes. Therefore, understanding the principles behind these transport phenomena is necessary for the targeted development of the tissue culture. To describe the internal actions of the cell in the tissue culture, as accurately as possible, biological and biochemical aspects, as well as engineering parameters such as internal and external mass transfer, fluid velocity, shear stress, have to be considered [8].

Due to the high complexity of such culture constructs, long lasting experimental techniques, modelling, and computational tools have to be combined to link different parameters to process conditions and critical process parameters (CPPs). If successful, this can allow a rapid evaluation of the product quality by means of Critical Quality Attributes (CQAs) [9]. Furthermore, modelling and computational tools can be integrated into Digital Twins (DTs), a virtual replica of a real process, service, or system [10]. DTs can help to anticipate potential issues and curb risks by testing new functionalities before rolling them out or they can be used in real‐time in parallel to the actual product for internal control. Hence, DTs are valuable tools for describing and understanding cellular reactions (e.g., mechanistic kinetic DTs), setting up meaningful and time‐saving experiments (e.g., digital twin‐assisted Design‐of‐Experiment), or designing and optimizing bioprocesses by digital twin‐based control strategies. This approach is already well established for cell‐based production of biopharmaceuticals [10, 11]. For tissue engineering, the potential has been recently uncovered [7, 12].

The results of both mathematical simulations and DTs are critically dependent on correct input parameters. Errors in input parameters can lead to dramatically wrong conclusions. However, acquiring accurate input parameters requires challenging techniques. Experimental techniques comprise analytic methods including on‐line and off‐line sensors have to be meticulously implemented. These techniques are used for characterization of culture systems (oxygen transfer, diffusion, permeation, flow, shear, mechanical load, etc.), and determination of cellular parameters (oxygen uptake rate, substrate uptake rate, metabolite production rates, etc.).

This review provides an overview, how transport processes within tissue cultures can be measured and summarizes state‐of‐the‐art techniques and remaining challenges for input generation to be used in comprehensive computational analysis.

## 2 RELEVANT ASPECTS OF TRANSPORT PROCESSES IN TISSUE CULTURES

Tissue cultures—like many other systems that deal with cell cultivation—entail several phenomena in which transport processes, mainly mass and momentum transfer, represent a key element in tissue development and maintenance (an overview of respective techniques is given in Table 1). The mass transfer controls how nutrients and growth/stimulating factors are transported to the tissue, and how waste products are removed and cells function in the tissue.

**TABLE 1:** General overview on techniques for organotypic tissue culture (adapted from [7])

Application	Techniques and devices
Cell expansion (2D, monolayer)	Well‐plates Flasks/roller bottles/multi‐tray systems Microcarrier for suspension culture (spinner, shake flask, stirred bioreactor)
Tissue generation and maturation (3D)	Non‐scaffold‐based (e.g., hanging drop microplates, spheroid microplates, magnetic levitation, Multi‐Organ‐Chips, aggregate culture in suspension culture) Scaffold‐based, for example, synthetic and biological macroporous carriers, hydrogels or de‐cellularized matrices (e.g., micropatterned surface microplates, transwelll membrane systems, Multi‐Organ‐Chips (“micro‐bioreactors”), tissue specific bioreactors (“macro‐bioreactors”))

To create a functional tissue, the maintenance of mass transport and the precise and reproducible control of environmental conditions are essential [1]. Cells, especially stem cells, are known respond to a variety of regulatory peptides/proteins in different developmental stages (e.g., differentiation, proliferation), different fate decisions (e.g., self‐renewal vs. lineage specification) as wells as to gradients of biomolecular concentrations and cell–cell and/or cell–matrix interactions (for references see [7]). These regulatory proteins are also produced and secreted by the cells, generating feedback loops. The required tissue architecture to allow for these regulatory loops can be achieved by scaffold‐free or scaffold‐based techniques. Scaffolds are especially used in more sophisticated bioreactor systems (reviewed in [7]), which create an appropriate biomimetic microenvironment, recapitulate tissue‐specific cell–cell contacts or cell–matrix interactions, mimic gradients of oxygen, nutrients, waste products or pH, and modulate diffusion of regulatory molecules. One of the major challenges in this regard is controlling the diffusion rates of growth factors, proteins, and other molecules for cell regulation and a careful exchange of nutrients and gases under spatial control [13, 14, 15].

Besides mass transfer, other transport processes like momentum and fluid flow are also important as they influence cell development. Shear stress below detrimental levels stimulates cells mechanically and can affect cell differentiation and proliferation [16, 17]. Compared to static conditions, perfusion can, for instance, enhance calcium and collagen production [18, 19], or increase mineralized matrix production by bone marrow cells [20, 21, 22] while decreasing chondrocyte‐driven cartilage production [23, 24]. Mullender et al. [25] demonstrated that the perfusion rate can change cell responsiveness as well. Furthermore, it was shown that the cell response to gradients of shear stress differs from that of the magnitude of shear stress [26, 27], as well as temporal and spatial variations [28]. Therefore, it is necessary to determine the precise shear stress stimuli that the cells actually experience to understand complex cell responses to it.

For appropriate design and operation of organotypic tissue cultures, not only a profound biological understanding of the tissue/organ of interest is required, but also an extensive “engineering” knowledge is necessary on special materials that enhance cell immobilisation (biocompatibility, biodegradability), special culture systems (bioreactors), including flow dynamics, mass transfer [29], shear effects (as discussed above), as well as control and cultivation strategies [8]. This creates diverse intercellular signaling networks and a complicated process, which is also manifested in in vitro culture of tissue constructs, most of them comprise dynamic non‐linear interplays between several components. This makes it very difficult to distinguish individual mechanisms in order to control the local microenvironment and the functional development of the tissue construct.

## 3 COMPUTATIONAL METHODS

Due to the high complexity of the microenvironment and flow dynamics, especially in micro‐bioreactors, it is very difficult to design controlled experiments. Computational models are therefore valuable tools to gain a comprehensive understanding of this complex biological systems. *In silico* models and DTs of the real system can be used for cell culture system design (bioreactors), simulation and analysis of mass transfer (nutrient supply, removal of metabolites deposits). Furthermore, this can also result in replacement or reduction of in vivo experiments complying with the reduction, refinement, and replacement principles [4, 30–33].

Developing an *in silico* model often starts with general formulations and interpretations of previous experiments and hypotheses, intending to figure out fundamental mechanisms and their interactions. The more key parameters and important partial processes can be identified the more representative the model setup comprises of. The outcome is a set of equations that describe how the quantity of interest (e.g., metabolite concentration) changes with time and environmental process parameters (e.g., temperature and pH). Different mechanisms (e.g., physicochemical phenomena) can then be studied to design or optimize tissue engineering bioreactors. Furthermore, mathematical modelling and computational tools can be used to discover the interconnection of complex regulatory processes, investigate the systematic influence of perturbations for optimal engineering design, develop new hypotheses, and ultimately evaluate the functionality of specific molecules for therapeutic applications [34, 35]. The simulation approach provides us with an opportunity to probe different scenarios and perform parametric studies of the hypothesized model. The results of such computational experiments are then compared to known experimental facts for validation of the model. Afterwards, the validated model may be used to optimize certain aspects of the studied system. Some demonstrated examples of this approach relevant to tissue engineering include optimizing construct and scaffold geometries as well as fluid flow patterns in bioreactors [36, 37, 38, 39]. Additionally, dynamic modelling can be a predictive tool for improving experimental design protocols.

Since different affecting variables, such as shear stress, are nearly impossible to measure due to the complex geometry of bioreactors, computational fluid dynamics (CFDs) emerged as a remedy. CFD can simulate fluid flow in freely definable scaffolds and bioreactor geometries [8, 40–44]. Given enough computer resources, high‐resolution simulation on micro‐computed tomography (μCT) reconstructed geometries can achieve precise flow distribution throughout the microstructure [45, 46]. Computational modelling is becoming the core of the development of the next generation of bioreactors and scaffolds, as it allows an enhanced combined consideration of biological and engineering effects for characterization of the system and the cellular response [9]. To complete the simulation using CFD, dynamic cellular parameters, such as cell growth and proliferation, are required to capture their effects on shear stress distribution. Different aspects that can be addressed by mathematical modelling of tissue cultures are reviewed in [7] and summarized in Table 2.

**TABLE 2:** Aspects addressed by mathematical modelling with respect to tissue culture (adapted from [7], for references see [7])

Aspects addressed by mathematical modelling	
Biological	Physico‐chemical
Cell growth^a^, morphology, heterogeneity, dynamics	Culture conditions^a^, cell handling, cell seeding
Cellular kinetics^a^	Biomechanical effects (e.g., shear stress)^a^
Signaling networks, regulatory networks, cell–cell and cell–matrix interactions	Penetration^a^, permeation^a^, diffusion^a^
Tissue specific effects	Fluid dynamics^a^, mass transfer^a^ (bioreactors, scaffolds)
Pharmacology	Chemical, physical and mechanical stimulation^a^

^a^ Related to computational analysis of transport processes.

In the field of computational biomechanics, the finite element method (FEM) is by far the most common numerical discretization and solution technique. The main strengths of FEM are its ability to work with complex geometries, its well‐developed mathematical background that provides a consistent way to treat material inhomogeneities and different constitutive equations for the material behavior, as well as the high availability of solvers [35, 47–49]. Although there are several examples of *in silico* studies, there are still challenges for using their full potential. One of the critical challenges of biological systems is the connection of simultaneous interrelated phenomena in different scales and unknown system importance. The ensuing multi‐disciplinarity makes it difficult to derive mathematical equations and find solutions. Therefore, multi‐scale methods are used to take care of individual cell interactions in micro‐scales and holistic behaviors in macro‐scales. There also exist meso‐scale methods, for example, Lattice Bolzman method (LBM) [50], which work in between the scales mentioned above [51, 52]. Due to the kinetic nature of the LBM, microscopic interactions in the system can be handled in porous media and other complex geometries [50]. Moreover, the inherently local dynamics used in LBM allow an efficient implementation to exploit the computational power of present and emerging supercomputing architectures [53]. Besides such algorithmic advantages, LBM shows high versatility for multi‐physics simulation and integration with tomographic measurement techniques as evident by works on perfusion flow in scaffold bioreactors [54] and even by patents for medical image analysis [55].

In general, model generations should follow Good Modelling Practice approaches and explicit workflows [56]. As a good example, the USA Food and Drug Administration (FDA) has provided standard technical guidelines [57] that need to be followed for digital tools to be used as medical devices. Several additional steps are necessary to utilize the model as a digital tool: verification, validation, and uncertainty quantifications [12]. Here, verification refers to the agreement between the results and the mathematical formulations, while validation is the establishing agreement with the experimental results. Uncertainty quantification should also be applied to make sure that the method is based on correct assumptions, is built correctly, and has already considered the effect of input uncertainty on the results. Other remaining challenges in the widespread application of *in silico* models in bioprocess technologies include; user‐friendliness of tools and user acceptance, precise documentation for a unified process modelling implementation, and generation of valid input variables for in silico models. The latter has been surprisingly overlooked in research as will be highlighted in Section 4, most likely due to the common misconception that the iterative nature of mathematical models can eventually generate accurate results. Therefore, there is an ever‐increasing necessity to emphasize and discuss different techniques for precise data generation.

## 4 TECHNIQUES FOR GENERATION OF INPUT VARIABLES FOR COMPUTATIONAL MODELS

### 4.1 Sensor techniques

Sensors are essential tools for marker‐free, continuous, on‐line or at‐line monitoring and control of cell metabolism and microenvironment parameters (e.g., temperature, pH, pressure, fluid flow, oxygen tension, metabolites, impedance, regulatory molecules, short‐lived reactive species, shear stress, electrical pacing, mechanical properties). They deliver information beyond microscopy techniques and end‐point tests [58, 59, 60], and have to be ‘able to detect events or changes in their environment at low concentrations under a complex matrix, which also permits multi‐parametric analyses at the same time with continuous monitoring and fast response at low cost’ [61].

Whereas these techniques help to investigate tissue cultures in the first place, they are indispensable in standardizing techniques that are routinely required for CQAs. Furthermore, they provide the data required for the determination of cellular metabolic parameters (Section 4.2) and physicochemical process parameters for the characterization of culture systems (Section 4.3). Therefore, sensor readings are closely coupled and correspond with good mathematical modelling practices of fluid dynamics and mass transfer effects in culture devices (Section 3). An overview of sensor techniques is given in Table 3. For a detailed explanation of the working principles, pros and cons, and specific applications, see [58, 59, 62, 63].

**TABLE 3:** Overview on sensor techniques for tissue culture devices (adapted from [58, 62], modified)

Parameter	Sensor type
*Cell culture supernatant*	
Oxygen	Direct amperometric sensors Clark‐type sensors Optical sensors (luminescence/fluorescence)
pH	Light addressable potentiometric sensors (LAPS) Ion‐selective field effect transistors (ISFET) Metal oxide‐based potentiometric sensors Optical sensors (lumenscence/fluorescence)
Glucose	Electrochemical (enzyme based) biosensors Optical biosensors Electrochemical multi‐electrode arrays (MEA)
Lactate	Electrochemical (enzyme based) biosensors Optical biosensors
Glutamate	Electrochemical (enzyme based) biosensors
Pyruvate	Electrochemical (enzyme based) biosensors
Reactive oxygen (ROS) and reactive nitrogen species (RNS)	Microsensor approaches in combination with electrochemical sensing techniques (ultramicroelectrode (UME) arrays, planar sputtered gold/cytochrome c electrodes, pyramid‐shaped Pt tip electrodes)
Proteins	Immunosensors
*Cellular properties*	
Cell density, cell morphology, marker secretion	Electric Cell‐Substrate Impedance Sensing (IDEs (Interdigitated Electrodes)), FETs (field‐effect transistor), ISFETS (ion‐sensitive field‐effect transistor (ISFET)
Tissue morphogenesis and maturation	Pillar deformation (fluorescence microscopy)
Mechanical strain of cellular barrier	Resistivity changes in impedimetric coplanar electrodes.
Barrier function and integrity, tight junction formation, cell growth and differentiation	Transepithelial/transendothelial electrical resistance (TEER) measurements
Barrier integrity	Transconductance measurements
Myokine secretion	Myokine concentration measurement by functionalized gold electrodes
Cardiac beat rate	Multi‐electrode array and atomic force microscopy measurements Voltage and displacement current measurement by large area electrodes Cantilever array

In recent years, the focus has been on integrating sensors in microfluidic systems (e.g., multi‐organ chips) to access parameters of cellular metabolism as well as morphological effects [58, 60, 62, 64–65], so far limited to extracellular readings providing indirect information about the metabolic state of the cells [58]. Future directions can focus on characterization of complex cell–cell and cell–matrix interactions and real‐time quantification of small molecules uptake and release as well as protein expression, as these are essential input parameters for computational models (compare Section 4.2) [58, 59]. Furthermore, probing and monitoring of relevant key organ functions using tissue‐/organ‐related biosensing strategies has to be established [62]. Limitations that have to be overcome for establishing routine applications include high complexity of fabrication technology and operation, as well as incompatibility with standard procedures [58, 59, 65, 66].

### 4.2 Cellular metabolic parameters relevant for modelling

Cell‐specific metabolic parameters such as uptake rates for oxygen and substrates, production rates for metabolites and carbon dioxide, as well as growth and death rates, are essential variables for computational models addressing fluid dynamics, mass transfer effects and metabolic activities in tissue cultures. Whereas methods for determining such variables are well‐established for biopharmaceutical cell culture using mostly established cell lines [57], this is hardly the case for tissue cultures for several reasons. An essential difference is that tissue cultures are mostly generated from primary cells and not well‐established cell lines. For the latter (e.g., Chinese Hamster Ovary [CHO] cells), a stable metabolism can be assumed over a large number of passages. Furthermore, most of these cell lines have been adapted to growth in suspension. Therefore, a sufficient number of cells can be produced to perform experiments under appropriate, reproducible culture conditions, for example, in well‐controlled bioreactor systems. Hence, it is possible to determine reliable data and investigate the impact of culture conditions on these parameters, for example, the relationship between metabolic rates and concentrations of oxygen, substrates or metabolites. From such data, thorough metabolic models can be formulated [67].

In the case of primary cells, however, determination of reliable data is more challenging. In most cases, just a small number of primary cells can be obtained from a biopsy of interest. Protocols for the proliferation of tissue cells have mainly been established only for adherent cells. This, in principle, allows to generate a large number of cells within several passages, even though it makes cultivation in controlled bioreactors a lot more challenging. More importantly, cellular properties and metabolic activities change significantly during the first passages. Therefore, data generated for cells in a higher passage number hardly reflect freshly isolated primary cells or even cells in vivo. Furthermore, cellular and metabolic parameters are donor‐specific [68, 69, 70, 71]. Due to the problems described for isolated primary cells, cell‐specific metabolic parameters have often been determined using immortalized cell lines such HepG2, HepaRG, HaCaT, hMSC‐tert cells, etc. (see below).

To make matters worse, tissue engineering is often aimed to obtain functional organotypic tissues that consist of several cell types within an appropriate extracellular matrix (3D, organoid, etc.). Under such conditions, cellular properties and metabolic activities are different to those in monolayer flask cultures. Owing to the difficulty of detecting the exact cell number required to calculate cell‐specific parameters in 3D or organoid cultures, cell‐specific metabolic parameters can often be determined as volume‐specific parameters only. For determination of volumetric rates, for example, in organoid cultures or multi‐organ chips, specific methods have to be established due to the small volumes [8]. Furthermore, gradients of, for example, oxygen or substrates/metabolites, have to be foreseen, making it more difficult to investigate the impact of culture conditions on these parameters. Often, these variables are determined as adaptation parameters in mathematical models for flow/metabolism (see below).

In the following example, aspects related to the determination of cell‐specific metabolic parameters are explicitly examined to determine cell‐specific oxygen uptake rates. Further metabolic rates are only considered briefly.

Oxygen has a tremendous impact on the cellular and metabolic activity of cells. Therefore, it is essential to generate an appropriate microenvironment with regard to cell‐specific oxygen concentrations. For example, in cartilage, liver or stem cells, lower oxygen concentrations seem to be advantageous with respect to the metabolic activity [72, 73]. With respect to mass transfer effects within 3D or organoid cultures, oxygen is usually regarded as more relevant than other medium compounds such as glucose or glutamine. This is often attributed to the poor solubility of oxygen in the culture medium, which restricts the diffusive penetration depth of oxygen within tissues in vivo in the range of only 100–200 μm [8]. In vitro, this is even worse, as artificially generated tissues are usually not vascularized, calling for anticipating severe oxygen gradients or even oxygen limitations. Therefore, the oxygen concentration adjustment is a critical matter in the design of any tissue culture system. But as exact sensory determination of the oxygen concentration within tissue constructs is still difficult and not well‐established (compare Section 2), oxygen profiles can often only be estimated through mathematical modelling, which itself depends on reliable values for cell‐specific oxygen uptake rates.

Methods used for the determination of parameters related to oxygen consumption are summarized in Table 4. These are mostly related to liver/HepG2 cells, due to the intensive research on cell‐based extracorporeal liver support systems and oxygen distribution within the respective bioreactors. Selected examples for other tissue types have been added. The values themselves are deliberately not provided, as the focus of this review is on the respective methods. Furthermore, values reported in the literature often show a considerable deviation. As an example, for the uptake rate of liver cells, reported values fluctuate by a factor of 25–30 [74]. This is probably due to different methods and environmental conditions (cell density, growth phase, substrate concentrations, 2D/3D) that have been applied. Most references referring to bioreactors have applied macro‐bioreactors. Only a few studies used the terminology to refer to micro‐bioreactors or organoid cultures, even though techniques for monitoring and controlling oxygen within such systems have been described [75, 76, 77, 78, 79].

**TABLE 4:** Comparison of methods used for determination of parameters related to oxygen consumption based on suggestions by Weise et al. [74], extended; ‘‘*n*’’: Specification of the number of samples, if this is evident from the publication

Refs.	purpose	Cell type	Method for determination of OUR and/or *k*_m_	Verification	Comment
[124]	Kinetics of oxygen metabolism in brain and liver tissues	Isolated tissue (liver, brain)	Whole organ consumption (volume specific), comparison of different kinetics, parameter adaptation and calculation of cell‐specific rates	Mean values + standard deviation	The results imply that the apparent kinetics for oxygen consumption in tissue differs from the kinetics for mitochondrial suspensions.
[125]	Gradients of O_2_ concentration in hepatocytes	Isolated rat hepatocytes (primary)	Polarographic assay (compare [126]), measured 1 h after preparation	Mean values + standard deviation cell‐specific uptake rate: *n* = 6; *k*_m_: *n* = 3 – 20	Cell‐specific oxygen uptake rates and *k*_m_ determined
[127]	Oxygen uptake rates in cultured rat hepatocytes	Rat hepatocytes	OUR experimentally determined. Perfusion system based on T‐75 tissue culture flasks. DO determined at inlet and outlet flow. Calculation of OUR from a simple mass balance depending on flow rate. *k*_m_ n.d.	*t*‐test at a significance level of 0.05; experiments were performed twice with two to three measurements per experiment	OUR of hepatocytes in culture may vary depending on the phase of culture (i.e., early vs. late) and on the extracellular environment
[128]	Metabolic activity of Hep G2 cells and primary rat hepatocytes were compared during in vitro application of a gel entrapment bioartificial liver.	HepG2 Rat hepatocytes (primary)	DO determined at inlet and outlet flow of a modified hollow fibre bioreactor with cells embedded in gel. Calculation of OUR from a simple mass balance depending on flow rate. *k*_m_ n.d.	Mean values + standard deviation Hep G2: *n* = 4;, rat hepatocytes: *n* = 16	Data system specific
[129]	Method for determining oxygen consumption rates of static 2D‐cultures	Rat hepatocytes	Device to determine the dependence of OUR on oxygen partial pressure for anchorage‐dependent cells cultured in standard culture dishes.	Mean values + standard deviation *n* = 4–6	Proof‐of‐concept for the technique, further studies of the effect of factors such as extracellular matrix composition, metabolic substrate, and drugs on the dependence of OUR on oxygen partial pressure suggested
[130]	Techniques for measurement of oxygen consumption rates of hepatocytes during attachment and post‐attachment	HepG2 Rat hepatocytes	Oxystat apparatus for HepG2 cells immobilized on Cytodex 3 microcarriers, flow cell for rat hepatocytes immobilized on single collagen layers, two‐compartment oxystat for rat hepatocytes during the attachment phase.	n.d.	OUR decreased with increasing cell density. The OUR measured for rat hepatocytes during and post‐attachment are significantly higher than those reported elsewhere (compare e.g., [74])
[131]	Oxygen uptake rate (OUR) of hepatocytes as parameter for the design of bioartificial liver assist (BAL) devices.	Porcine hepatocytes	Special culture chamber for monolayer culture with integrated DO‐sensors. Measured oxygen tension versus time, parametric data was processed off‐line to generate OUR versus oxygen tension curves.	Mean values + standard deviation *n* = 12	Examination of the OUR_max_ and *k*_m_ revealed different cellular metabolic states for the initial attachment phase and the subsequent steady‐state culture condition.
[132]	Investigation of oxygen transfer to cultured hepatocytes in microchannel parallel‐plate bioreactors with and without an internal membrane oxygenator with a mathematical model	Rat hepatocytes	Model calculations for different values of Pe and Da‐number. Da includes volume specific oxygen uptake rate. Cell‐specific oxygen uptake rate not given. *k*_m_ taken from [129]	Results were confirmed by fluorescence imaging experiments.	Model calculation verified qualitatively.
[133]	Investigation on the role of oxygen in modulating cellular functions under steady state oxygen gradients in cell culture.	Rat hepatocyte (primary)	Model of oxygen transport in a flat‐plate reactor to estimate oxygen distribution at the cell surface. Modelling parameters for OUR_max_ and *k*_m_ taken from [129]	Experimental measurements of outlet oxygen concentration from various flow conditions were used to validate model predictions	Model calculation verified quantitatively.
[94]	Development of a method for determining oxygen consumption rates of static cultures from microplate measurements of pericellular dissolved oxygen concentration	Primary rat hepatocytes	Protocol based on a non‐invasive oxygen‐sensing microplate and a simple mathematical model derived from Fick's Law. Data for a large number of mammalian cells and microbes. *k*_m_ n.d.	Cell‐specific oxygen uptake rate determined from measured volumetric oxygen uptake rate and cell density. *n* = 5 for method development, standard deviation indicated graphically	Method development. OUR data for hepatocytes compared to own studies (not given)
[103]	Numerical model for investigation of fluid flow and oxygen transport and consumption in the AMC Bioartificial Liver	Hepatocytes, not specific	Values for OUR and *k*_m_ taken from [131]. Those were determined for 2‐D culture	Not verified experimentally.	Parameter studies for different configuration
[74]	Analysis and comparison of oxygen consumption of HepG2 cells in a monolayer and three‐dimensional high density cell culture	HepG2	Measurement of DO by optical sensors in 2D culture and within a hydrogel Model for oxygen transfer and consumption	Model validation by experimental data	Comparison of evaluated data in the literature with own measurement data
[99]	Analysis of oxygen transport in microfluidic bioreactors	Intended for culture of hepatocytes and cardiomyocytes	Mathematical model to determine oxygen transport within microchannel parallel plate bioreactor with and without an internal oxygen‐permeable membrane was developed. Oxygen diffusion in both directions, including parallel to the flow direction and perpendicular to the flow direction due to the cellular oxygen uptake were considered. OUR_max_ from [127], *k*_m_ neglected	Model not verified experimentally. No sensitivity analysis regarding OUR_max_.	Values for OUR_max_ have been experimentally determined for a different culture system.
[100]	Investigating oxygen transport efficiencies in precision‑cut liver slice‑based organ‑on‑a‑chip devices	Mouse liver slices	The transport of nutrients in the simulation was described by the generic diffusion. The consumption of oxygen was calculated with Michaelis–Menten reaction kinetics. Furthermore, to account for cellular processes such as necrosis, a factor was implemented to eliminate the reaction term for the cellular consumption by going to zero below a critical DO. OUR_max_ and *k*_m_ taken from [124]	Model not verified experimentally. No sensitivity analysis regarding OUR_max_.	The optimal device architecture derived from the modelling was then fabricated and its operation confirmed with an LDH assay.
[80]	Computer modelling of the oxygen supply and demand of cartilage cells	Isolated chicken chondrocytes	OUR experimentally determined and plotted versus DO, parameter estimation for Michaelis‐Menten‐kinetic	Qualitatively discussed	OUR decreases at low oxygen tensions. The metabolism of chondrocytes is not controlled simply by the available oxygen supply [134].
[135]	Modelling of oxygen uptake with three‐dimensional chondrocyte pellets	Cartilage cells from mini‐pig (knee and elbow)	Oxygen uptake rate of a single pellet measured in a special designed reaction chamber. Mass balance equations for determination of oxygen profile in chondrocyte pellets	Diffusion coefficient as fitting parameter	Cell‐specific oxygen uptake rate determined for suspended chondrocytes
[136]	Real‐time monitoring of specific oxygen uptake rates in a microfluidic cell culture device	Embryonic stem cells	Monitoring of cell growth from phase contrast microscopy images, and of respiration using optical sensors for dissolved oxygen connected to inlet and outlet of the microfluidic culture device. **Calculation of OUR from a simple mass balance depending on flow rate**.	n.d.	Label‐free and real‐time monitoring of oxygen concentrations and cell proliferation

In respect to the relationship between the volumetric oxygen uptake rate and the oxygen concentration most studies cited in Table 4 refer to the work of Haselgrove et al. in 1993 [80], where an equation following Michaelis–Menten‐Kinetics was used:

(1) $$\mathrm{OUR}=q_{\mathrm{cell}}\cdot N_{\mathrm{cell}}\cdot \frac{c_{O_{2}}}{k_{m}+c_{O_{2}}}$$

OUR: oxygen uptake rate (mol L^–1^ h^–1^); *q*_cell_: cell‐specific oxygen uptake rate (mol cell^–1^ h^–1^); *N*_cell_: cell density (cells L^–1^); c_O2_: oxygen concentration (mol L^–1^); *k*_m_: Michaelis–Menten‐constant (mol L^–1^).

Even if the assumptions of Equation (1) are reasonable, actual verification is missing. As the exact determination of *N*_cell_ is often difficult for 3D culture, *q*_cell_ * *N*_cell_ has been determined as the volumetric parameter instead (mol L^–1^ h^–1^). This problem in particular applies to microfluidic cell culture devices with respect to real‐time monitoring of cell densities and oxygen concentration [77, 81].

With respect to cell‐specific metabolic rates as input parameters for growth, substrate consumption or metabolite production in mass transfer models, mostly glucose as the substrate and lactate as the metabolite are considered, with some focus on the relevance of other compounds such as glutamine, ammonia or carbon dioxide [8]. Determination of cell‐specific substrate uptake and lactate production rates is usually based on time‐dependent analysis of the concentrations of the respective compounds, as well as the cell numbers. The latter can further be used for calculation of cell‐specific growth rates.

Even though the relevance of the abovementioned parameters and their tremendous impact on culture conditions is well known from established cell lines, extensive studies on tissue cells addressing these effects are rare. To some extent, such studies have been performed for monolayer cultures [82, 83], but hardly for 3D and organoid cultures. Techniques for determination (compare Section 2) and long‐term monitoring [75, 84] of glucose and lactate for such complex systems have been established, and challenges related to the precise 3D determination of cell number for organoid culture have been discussed before.

Several studies exist on modelling of cell growth, glucose uptake and lactate production in tissue cultures [37, 83, 85–90], in most of which values for cell‐specific growth, uptake and production rates were either taken from literature or determined by parameter estimation (similar to oxygen uptake rates; see above). Particularly, noteworthy in this context is the work of Higuera et al. [83], as they compared, measured and simulated values.

In conclusion, the above discussion reflects the problems in determination of reliable cell‐specific metabolic parameters. This has to be taken into account if these parameters are applied in mass transfer models. Likewise, sensitivity analyses should be included, and discussion on how required simplifications and assumptions in the modelling have an inevitable influence on the adjustment parameters.

### 4.3 Physico‐chemical process parameters relevant for modelling

Several engineering parameters are relevant for modelling of mass transfer effects and fluid dynamics in tissue culture. For transport mechanisms, oxygen transfer rate, permeability, and diffusion coefficients are addressed. Fluid flow is mainly influenced by shear distribution, scaffold structure, and porosity of the scaffold. Respective techniques will be discussed in the following.

#### 4.3.1 Oxygen transfer rates

As already highlighted in Section 4.2, oxygen transfer is a matter of utmost importance for tissue cultures due to the low solubility of oxygen in the culture medium and the tremendous impact of the oxygen concentration on the physiology of tissue cells [8, 76, 91, 92]. Naturally, the oxygen requirement of cells (volume‐specific oxygen uptake rate, OUR) must be in accordance with the oxygen supply (oxygen transfer rate; OTR). For aeration of tissue culture systems, one or a combination of the following methods can be applied, depending mainly on the size of the culture system: surface aeration, direct sparging, indirect and/or membrane aeration (diffusion), medium perfusion, increasing the partial pressure of oxygen, and increasing the atmospheric pressure [93]. Oxygen dissolved in the medium is mainly transported by diffusion within the bulk fluid phase, from the bulk to the surface of the organoid through the boundary layers, and subsequently reaches the bulk of the organoid. As the spatial distribution of oxygen within the boundary layer and within the bulk of the organoid can hardly be measured so far, mass transfer models that are applied here are based on diffusion (e.g., Fick's law).

Several techniques for determination of oxygen transfer rates are at hand in the case of surface aeration or direct sparging, which include the sulphite oxidation method, the dynamic method, the gassing out method (reviewed by [8]), and the membrane aeration [93] among others. Most of these methods have been developed and standardized for the stirred tank and similar bioreactors [74] or adapted to flask type cultures (well plates, T‐flask) or shake flasks with surface aeration [94].

The exact experimental determination of OTR for tissue culture systems (mainly micro‐bioreactors) has proven to be extremely difficult owing to the small size and the complexity of such systems, as well as the limited availability of techniques for measuring the oxygen concentration, as outlined in Section 4.1 [74, 95–98]. Hence, OTR is often considered within mass transfer models [46, 74, 92, 99–103].

#### 4.3.2 Diffusion/permeability coefficients

As mass transfer in in vitro tissue organoids relies mostly on diffusion/permeation, proper determination of the respective transport coefficients is important. Diffusion is a transport process in which molecules move via Brownian motion in a volume or area, driven by the concentration gradient in the direction from higher to lower concentrations. Mathematically, it is usually described by Fick's law [104, 105]. Permeation is merely an aspect of diffusion, and while diffusion is related to the movement of molecules in a system, permeation describes how fast molecules move through a system, and is characterized by the permeability coefficient. Both parameters depend on the molecular weight of the compound and the properties of the medium or tissue, among others [104].

Within mass transfer models, mostly low‐molecular‐weight compounds such as oxygen, glucose, and lactate are considered for diffusion within the bulk medium surrounding a tissue organoid and within the organoid. In most cases, diffusion coefficients determined for diffusion in water at medium temperatures are applied. For the bulk medium, this seems to be a sensible approach. For the tissue organoid itself, however, the respective properties should be considered. When hydrogels are applied as the scaffold material, this problem should be examined even more closely, as hydrogels consist of a water‐containing polymer network, where the material transport takes place predominantly in the aqueous phase. For hydrogels, the polymer network turns out to be the actual barrier for molecular movement. Decisive factors for the diffusion of substances through a hydrogel are the nature and density of the cross‐linked network and the shape and size of the particles. The water solubility and the surface charge also play major roles. In general, molecules migrate better and faster through a large‐meshed, flexible network than through a small‐meshed, rigid network. Likewise, molecules with a smaller hydrodynamic radius diffuse faster through hydrogels than large ones [106]. For larger molecules (approx. 4 kD), anomalous diffusion (subdiffusion) has to be expected, which cannot be described by Fick's law. This effect could be confirmed by Hsu et al. for in vivo skin models [107].

Aside from skin models/biopsies and cartilage, high molecular weight compounds such as growth factors or substances applied in penetration tests (e.g., skin models or drug testing) have hardly been considered in mass transfer models. Due to the high relevance of skin permeation following different mechanisms, mainly the transcellular, the intercellular and the follicular pathway [108], several methods have been developed to determine skin permeability, which will be summarized briefly in the following.

The Franz diffusion cell is a well‐known device to measure the permeation of a substance through a tissue. Due to the usual size (height) of Franz diffusion cells, they are well established for skin biopsies but can hardly be applied for small tissue constructs [109]. Fluorescence recovery after photobleaching (FRAP) is a method to measure molecular diffusion in tissues or gels, mainly for high molecular weight compounds. For this, the substance must be labelled with a fluorochrome. Mostly fluorescence‐labelled proteins or FITC‐dextrans (fluorescein isothiocyanate‐dextrans) with different molecular weight/sizes are used [110, 111]. Further imaging methods for the determination of diffusion of molecules in the skin are Fourier‐transform‐infrared (FTIR) spectroscopy, two‐photon fluorescence correlation spectroscopy in combination with fluorescence correlation spectroscopy (FCS) and optical coherence tomography (reviewed by [15]). These methods are non‐invasive and non‐destructive but require expensive equipment.

As mentioned before, most methods for determining diffusion and permeability parameters have been developed for large biopsies and can hardly be applied for small‐scale tissue cultures. But this is indispensable if either multi‐well test systems with several samples run in parallel or multi‐organ chips are applied. Furthermore, most methods require treatment of the sample in one or the other way. Therefore, it is quite difficult to determine the time‐depending changes of diffusion and permeation. Hsu et al. developed a method based on fluorescence measurements to determine and simulate the permeation of high molecular weight compounds through a gel matrix in a multi‐organ‐chip and applied this to in vivo cultivated skin models [15, 105, 112].

#### 4.3.3 Porosity

The porosity of the scaffold—defined as the sum of the volumes of the pores divided by the sum of the volume of the scaffold material—can influence mass transport effects within the scaffold and by this the biological functionality and mechanical durability and is therefore important to be evaluated. Assessment techniques include theoretical approaches (e.g., unit cube analysis and mass technique), scanning electron microscopy (SEM), mercury porosimetry, gas pycnometry, gas adsorption, and flow porosimetry, all of which are associated with several limitations and pitfalls. A lack of sensitivity to characterize inconsistent/irregular architectures, destructiveness, and the necessity to perform complementary experiments are some of the more common drawbacks of the aforementioned techniques (reviewed by [113] and [114]). On the other hand, micro‐CT has a key advantage over other approaches since it is a single, non‐destructive technique that can be used in high‐resolution modes to scrutinize the intricate micro‐architecture of scaffolds, even if they contain irregular spatial layouts.

#### 4.3.4 Scaffold permeability

Successful tissue regeneration depends on the scaffold's ability to simultaneously allow nutrient diffusion, waste removal and to provide a sufficient mechanical environment. This is particularly mandatory to promote regeneration of bone tissue, where bone scaffolds should have a set of properties in terms of pore size and distribution, porosity ratio, tortuosity, interconnectivity, and specific surface area, among others. However, many of these parameters are interdependent and exhibit complex or even ambiguous results when it comes to their relation to mass transport and the biological performance of the scaffold [115]. For instance, even though larger pore sizes and higher porosity ratios in scaffolds are often ascribed to improved bone growth, some studies have reported limited to no significant difference in bone regeneration capacity of scaffolds with varying porosity ratios, while the influence of pore size has been similarly a matter of debate [116].

To address these conflicting reports regarding the role of the design parameters, scaffold permeability has been introduced as a macroscopic parameter that indirectly accounts for all the aforementioned geometrical features. Methods of permeability evaluation are often classified into two general categories: experimental test benches and computational approaches (reviewed extensively in [117]). In experimental methods, the permeability is quantified in the system either by measuring the pressure drop under a constant flow rate or by measuring the volumetric flow rate for a given pressure difference applied to the scaffold. In purely viscous flows, that is, Darcy regime (negligible inertial losses; Reynolds number < 1), the permeability K can be calculated by Darcy's law:

(2) K=(QμL)/(AΔP)

In Equation (2), *Q* is the volumetric flow rate, Δ*P* is the pressure drop through the scaffold, *A* and *L* are the cross‐sectional area and the thickness/length of the scaffold in the direction of flow, respectively, and *μ* is the dynamic viscosity of the fluid. Here the impact of the individual molecule involved (size, charge, hydrophobicity) has to be considered as well. Additionally, permeability can be estimated indirectly by measuring geometrical features of the scaffold (e.g., pore size, porosity, tortuosity, etc.) and using semi‐empirical models such as the Ergun–Forchheimer correlation [117]; in particular, when the assumption of the Darcy regime cannot be met.

One main challenge in the experimental assessment of scaffold permeability is a lack of widely shared, inter‐laboratory protocols, making it nearly impossible to compare values obtained in different laboratories. Moreover, as the permeability evaluation is based on simultaneous measurement of multiple quantities (i.e., upstream and downstream pressure and flow rate), several sensors/transducers have to be implemented, which introduce inaccuracy to the system [118]. Critically, the sensitivity and the range width of these transducers have to be selected as a function of the permeability range. Another difficulty is raised from the fact that most experimental methods require high fluid pressures, large time intervals, and several cycles of measurement to ensure reproducible results [119, 120]. This can lead to irreversible deformation in the scaffold structure and induce obstruction of small pores, which in turn causes fluid entrapment inside the scaffold, another possible source of error.

An alternative way of determining the permeability coefficient of scaffolds is the computational simulation of fluid flow dynamics [116]. By numerically solving partial differential equations that describe the fluid motion in different flow regimes, the permeability of the scaffold can be computed. For simulation, the geometrical 3D models of the scaffold can either be created from computer‐aided design (CAD), or by image‐based techniques such as micro‐CT [121]. Since even small variations between the designed architecture and the manufactured scaffold can strongly influence the flow field [122], micro‐CT has been deemed the gold standard for generating realistic 3D models of scaffolds, as well as measuring their geometrical characteristics (e.g., pore size and porosity) as input parameters for iterative processes [123]. Nevertheless, insufficient spatial image resolution can influence the accuracy of the 3D model and increase the surface roughness, which in turn would cause an underestimation of the computed permeability coefficient. On a more fundamental level, making accurate boundary conditions at different scales as well as incorporating material properties is a crucial challenge of numerical simulations.

## 5 CONCLUDING REMARKS

The complexity of an in‐depth characterization of fluidics and transport processes within organotypic tissue cultures requires the combination of experimental techniques, modelling, and computational tools. Tissue culture techniques are well‐established, modelling tools are at hand, and sensor technologies are increasingly being integrated in culture devices. The main current problem is that mathematical simulations need valid material property values and correct input parameters, because errors in input values can lead to completely wrong predictions, even if the intermediate model is accurate. As outlined in this review, there are still many areas, where validated and generally accepted measurement methods for determination of the input parameters are lacking. In these cases, values are either taken from the literature, sometimes neglecting limitations of the original source, or the values result as adjustment parameters in the modelling, with the risk of generating self‐fulfilling prophecies. Care needs to be taken to critically discuss how realistic such values are. This includes discussion on how the required simplifications and assumptions in the modelling may influence such adjustment parameters. Furthermore, if the precision of input parameters is uncertain, sensitivity analyses would allow an estimation of the magnitude of possible effects. Following such Good Modelling Practice approaches when setting up and validating computational models will, therefore, lead to better estimations of the interesting complex processes inside organotypic tissue cultures in the future.

## NOMENCLATURE

OUR, oxygen uptake rate (mol L^–1^ h^–1^)

*q*_cell_, cell‐specific oxygen uptake rate (mol cell^–1^ h^–1^)

*N*_cell_, cell density (cells L^–1^)

c_O2_, oxygen concentration (mol L^–1^)

*k*_m_, Michaelis–Menten‐constant (mol L^–1^)

*K*, permeability (Da)

*Q*, volumetric flow rate (m^3^ s^–1^)

Δ*P*, pressure drops through the scaffold (Pa)

*A*, Cross‐sectional area (m^2^)

*L*, thickness/length of the scaffold in the direction of flow (m)

*μ*, dynamic viscosity of the fluid (Pa s)

## CONFLICT OF INTEREST

The authors have declared no conflicts of interest.

## AUTHOR CONTRIBUTIONS

Ehsan Fattahi, Ralf Pörtner, Arndt F. Schilling and Shahed Taheri conceived the idea and wrote the manuscript. Arndt F. Schilling and Thomas Becker revised and edited the manuscript. All authors have approved the final manuscript.

## Data Availability

Data sharing not applicable to this article as no datasets were generated or analysed during the current study.

## References

[1] Haycock JW . 3D cell culture: a review of current approaches and techniques. Methods Mol Biol. 2011;695:1‐15. 21042962 10.1007/978-1-60761-984-0_1

[2] Hayden PJ , Harbell JW . Special review series on 3D organotypic culture models: introduction and historical perspective. In Vitro Cell Dev Biol‐Animal. 2021;57:95‐103. 10.1007/s11626-020-00500-2 PMC7687207 33237402

[3] Dhaliwal A . Three dimensional cell culture: a review. Mater Methods. 2012;2:162.

[4] Doke SK , Dhawale SC . Alternatives to animal testing: a review. Saudi Pharm J. 2015;23:223‐229. 26106269 10.1016/j.jsps.2013.11.002 PMC4475840

[5] Langhans SA . Three‐dimensional in vitro cell culture models in drug discovery and drug repositioning. Front Pharmacol. 2018;9:6. 29410625 10.3389/fphar.2018.00006 PMC5787088

[6] Liu J , Zheng H , Dai X , Poh P , Machens HG , Schilling A F . Transparent PDMS bioreactors for the fabrication and analysis of multi‐layer pre‐vascularized hydrogels under continuous perfusion. Front Bioeng Biotechnol. 2020;8:10.3389/fbioe.2020.568934 PMC7785876 33425863

[7] Möller J , Pörtner R . Digital twins for tissue culture techniques—concepts, expectations, and state of the art. Processes. 2021;9:447.

[8] Salehi‐Nik N , Amoabediny G , Pouran B , et al. Engineering parameters in bioreactor's design: a critical aspect in tissue engineering. Biomed Res Int. 2013;2013:762132. 24000327 10.1155/2013/762132 PMC3755438

[9] Geris L . In Vivo, In Vitro, In Silico: Computational Tools for Product and Process Design in Tissue Engineering. In: Computational Modeling in Tissue Engineering. Studies in Mechanobiology, Tissue Engineering and Biomaterials. 10 Springer Berlin Heidelberg; 2013; pp. 1–15.

[10] Herwig C , Pörtner R , Möller J . *Digital Twins: tools and concepts for smart biomanufacturing* . Advances in Biochemical Engineering/Biotechnology (1st ed.). Springer International Publishing; 2021. Imprint: Springer, Cham.

[11] Herwig C , Pörtner R , Möller J . *Digital Twins: applications to the design and optimization of bioprocesses* . Advances in Biochemical Engineering/Biotechnology (1st ed.). Springer International Publishing; 2021. Imprint: Springer, Cham.

[12] Geris L , Lambrechts T , Carlier A , Papantoniou I . The future is digital: in silico tissue engineering. Curr Opin Biomed Eng. 2018;6:92‐98.

[13] Chung CA , Yang CW , Chen CW . Analysis of cell growth and diffusion in a scaffold for cartilage tissue engineering. Biotechnol Bioeng. 2006;94:1138‐1146. 16586509 10.1002/bit.20944

[14] Garzón‐Alvarado DA , Velasco MA , Narváez‐Tovar CA . Modeling porous scaffold microstructure by a reaction‐diffusion system and its degradation by hydrolysis. Comput Biol Med. 2012;42:147‐155. 22136697 10.1016/j.compbiomed.2011.11.002

[15] Hsu H‐H , Schimek K , Marx U , Pörtner R . Measurement and simulation of permeation and diffusion in native and cultivated tissue constructs: 10. In: Dobrzański LA , ed. Biomaterials in Regenerative Medicine. InTech; 2018.

[16] Kreke MR , Huckle WR , Goldstein AS . Fluid flow stimulates expression of osteopontin and bone sialoprotein by bone marrow stromal cells in a temporally dependent manner. Bone. 2005;36:1047‐1055. 15869916 10.1016/j.bone.2005.03.008

[17] Jossen V , Pörtner R , Kaiser SC . Mass production of mesenchymal stem cells—impact of bioreactor design and flow conditions on proliferation and differentiation. In: Eberli D , ed. Cells and Biomaterials in Regenerative Medicine. InTech; 2014.

[18] Gomes ME , Sikavitsas VI , Behravesh E , et al. Effect of flow perfusion on the osteogenic differentiation of bone marrow stromal cells cultured on starch‐based three‐dimensional scaffolds. J Biomed Mater Res A. 2003;67:87‐95. 14517865 10.1002/jbm.a.10075

[19] Mizuno S , Allemann F , Glowacki J . Effects of medium perfusion on matrix production by bovine chondrocytes in three‐dimensional collagen sponges. J Biomed Mater Res. 2001;56:368‐375. 11372054 10.1002/1097-4636(20010905)56:3<368::aid-jbm1105>3.0.co;2-v

[20] O'Dea RD , Byrne HM , Waters SL . Continuum modelling of in vitro tissue engineering: a review. In: Geris L , ed. *Computational Modeling in Tissue Engineering*. *Studies in Mechanobiology, Tissue Engineering and Biomaterials* . Springer Berlin Heidelberg; 2013:229‐266.

[21] Yu X , Botchwey EA , Levine EM , et al. Bioreactor‐based bone tissue engineering: the influence of dynamic flow on osteoblast phenotypic expression and matrix mineralization. Proc Natl Acad Sci USA. 2004;101:11203‐11208. 15277663 10.1073/pnas.0402532101 PMC509184

[22] Cartmell SH , Porter BD , García AJ , Guldberg RE . Effects of medium perfusion rate on cell‐seeded three‐dimensional bone constructs in vitro. Tissue Eng. 2003;9:1197‐1203. 14670107 10.1089/10763270360728107

[23] Raimondi MT , Moretti M , Cioffi M , et al. The effect of hydrodynamic shear on 3D engineered chondrocyte systems subject to direct perfusion. Biorheology. 2006;43:215‐222. 16912395

[24] Raimondi MT , Bonacina E , Candiani G , et al. Comparative chondrogenesis of human cells in a 3D integrated experimental‐computational mechanobiology model. Biomech Model Mechanobiol. 2011;10:259‐268. 20549292 10.1007/s10237-010-0232-8

[25] Mullender M , El Haj AJ , Yang Y , et al. Mechanotransduction of bone cells in vitro: mechanobiology of bone tissue. Med Biol Eng Comput. 2004;42:14‐21. 14977218 10.1007/BF02351006

[26] Bao X , Lu C , Frangos JA . Temporal gradient in shear but not steady shear stress induces PDGF‐A and MCP‐1 expression in endothelial cells: role of NO, NF kappa B, and egr‐1. ATVB. 1999;19:996‐1003. 10.1161/01.atv.19.4.996 10195928

[27] Davies PF , Remuzzi A , Gordon EJ , et al. Turbulent fluid shear stress induces vascular endothelial cell turnover in vitro. Proc Natl Acad Sci USA. 1986;83:2114‐2117. 3457378 10.1073/pnas.83.7.2114 PMC323241

[28] Davies PF , Barbee KA , Volin MV , Robotewskyj A , et al. Spatial relationships in early signaling events of flow‐mediated endothelial mechanotransduction. Annu Rev Physiol. 1997;59:527‐549. 9074776 10.1146/annurev.physiol.59.1.527

[29] Kuo JS , Chiu DT . Controlling mass transport in microfluidic devices. Annu Rev Anal Chem (Palo Alto Calif). 2011;4:275‐296. 21456968 10.1146/annurev-anchem-061010-113926 PMC5724977

[30] Ingber DE , van Mow C , Butler D , et al. Tissue engineering and developmental biology: going biomimetic. Tissue Eng. 2006;12:3265‐3283. 17518669 10.1089/ten.2006.12.3265

[31] Lang A . In silico methods – computational alternatives to animal testing. ALTEX. 2018:126‐128. 10.14573/altex.1712031 29374440

[32] Madden JC , Enoch SJ , Paini A , Cronin MT , Review of in a. Silico tools as alternatives to animal testing: principles, resources and applications. Alternatives to Laboratory Animals: ATLA. 2020;48:146‐172. 33119417 10.1177/0261192920965977

[33] Passini E , Britton OJ , Lu HR , et al. Human in silico drug trials demonstrate higher accuracy than animal models in predicting clinical pro‐arrhythmic cardiotoxicity. Front Physiol. 2017;8. 10.3389/fphys.2017.00668 PMC5601077 28955244

[34] Fischer HP . Mathematical modeling of complex biological systems: from parts lists to understanding systems behavior. Alcohol Res Health. 2008;31:49‐59. 23584751 PMC3860444

[35] Maas SA , Ellis BJ , Ateshian GA , Weiss JA . FEBio: finite elements for biomechanics. J Biomech Eng. 2012;134:11005. 10.1115/1.4005694 PMC3705975 22482660

[36] Freitas D , Almeida HA , Bártolo PJ . Perfusion bioreactor fluid flow optimization. Proc Technol. 2014;16:1238‐1247.

[37] Hossain MS , Bergstrom DJ , Chen XB . Modelling and simulation of the chondrocyte cell growth, glucose consumption and lactate production within a porous tissue scaffold inside a perfusion bioreactor. Biotechnol Rep. 2015;5:55‐62. 10.1016/j.btre.2014.12.002 PMC5466199 28626683

[38] Nims RJ , Cigan AD , Albro MB , et al. Matrix production in large engineered cartilage constructs is enhanced by nutrient channels and excess media supply. Tissue Eng Part C Methods. 2015;21:747‐757. 25526931 10.1089/ten.tec.2014.0451 PMC4499772

[39] Hussein MA , Esterl S , Pörtner R , et al. Impulse and mass transport in a cartilage bioreactor using the lattice Boltzmann method. Int J Comut Fluid Dyn. 2008;22:341‐350.

[40] Mokhtari‐Jafari F , Amoabediny G , Haghighipour N , et al. Mathematical modeling of cell growth in a 3D scaffold and validation of static and dynamic cultures. Eng Life Sci. 2016;16:290‐298.

[41] Jungreuthmayer C , Jaasma MJ , Al‐Munajjed AA , et al. Deformation simulation of cells seeded on a collagen‐GAG scaffold in a flow perfusion bioreactor using a sequential 3D CFD‐elastostatics model. Med Eng Phys. 2009;31:420‐427. 19109048 10.1016/j.medengphy.2008.11.003

[42] Jungreuthmayer C , Donahue SW , Jaasma MJ , et al. A comparative study of shear stresses in collagen‐glycosaminoglycan and calcium phosphate scaffolds in bone tissue‐engineering bioreactors. Tissue Eng Part A. 2009;15:1141‐1149. 18831686 10.1089/ten.tea.2008.0204

[43] Williams KA , Saini S , Wick TM . Computational fluid dynamics modeling of steady‐state momentum and mass transport in a bioreactor for cartilage tissue engineering. Biotechnol Prog. 2002; 18:951‐963. 12363345 10.1021/bp020087n

[44] Sucosky P , Osorio DF , Brown JB , Neitzel GP . Fluid mechanics of a spinner‐flask bioreactor. Biotechnol Bioeng. 2004;85:34‐46. 14705010 10.1002/bit.10788

[45] Cioffi M , Boschetti F , Raimondi MT , Dubini G . Modeling evaluation of the fluid‐dynamic microenvironment in tissue‐engineered constructs: a micro‐CT based model. Biotechnol Bioeng. 2006;93:500‐510. 16224789 10.1002/bit.20740

[46] Cioffi M , Küffer J , Ströbel S , et al. Computational evaluation of oxygen and shear stress distributions in 3D perfusion culture systems: macro‐scale and micro‐structured models. J Biomech. 2008;41:2918‐2925. 18789444 10.1016/j.jbiomech.2008.07.023

[47] Ateshian GA , Maas S , Weiss JA . Multiphasic finite element framework for modeling hydrated mixtures with multiple neutral and charged solutes. J Biomech Eng. 2013;135:111001. 23775399 10.1115/1.4024823 PMC3792408

[48] Ateshian GA , Nims RJ , Maas S , Weiss JA . Computational modeling of chemical reactions and interstitial growth and remodeling involving charged solutes and solid‐bound molecules. Biomech Model Mechanobiol. 2014;13:1105‐1120. 24558059 10.1007/s10237-014-0560-1 PMC4141041

[49] Ateshian GA , Shim JJ , Maas SA , Weiss JA . Finite element framework for computational fluid dynamics in FEBio. J Biomech Eng. 2018;140:21001. 10.1115/1.4038716 PMC5816258 29238817

[50] Fattahi E , Waluga C , Wohlmuth B , et al. Lattice Boltzmann methods in porous media simulations: from laminar to turbulent flow. Comput Fluids. 2016;140:247‐259.

[51] Hussein MA , Esterl S , Pörtner R , et al. On the lattice Boltzmann method simulation of a two‐phase flow bioreactor for artificially grown cartilage cells. J Biomech. 2008;41:3455‐3461. 19019373 10.1016/j.jbiomech.2008.09.034

[52] Sayed M , A A , Hussein MA , Becker T . An innovative lattice Boltzmann model for simulating Michaelis‐Menten‐based diffusion‐advection kinetics and its application within a cartilage cell bioreactor. Biomech Model Mechanobiol. 2010;9:141‐151. 19633990 10.1007/s10237-009-0164-3

[53] Fattahi E , Waluga C , Wohlmuth B , Rüde U . Large scale lattice Boltzmann simulation for the coupling of free and porous media flow. In: Kozubek T , Blaheta R , Šístek J , et al., eds. High Performance Computing in Science and Engineering: Second International Conference, HPCSE 2015, Soláň, Czech Republic, May 25–28, 2015, Revised Selected Papers, Springer International Publishing, Cham 2016; pp. 1‐18.

[54] Spencer TJ , Hidalgo‐Bastida LA , Cartmell SH , et al. In silico multi‐scale model of transport and dynamic seeding in a bone tissue engineering perfusion bioreactor. Biotechnol Bioeng. 2013;110:1221‐1230. 23124479 10.1002/bit.24777

[55] Bernhardt D , Scheuering M , Vega‐Higuera F Device and computed tomography scanner for determining and visualizing the perfusion of the myocardial muscle. US9323887B2, United States Patent and Trademark Office, 26 March 2016.

[56] Kroll P , Hofer A , Stelzer IV , Herwig C . Workflow to set up substantial target‐oriented mechanistic process models in bioprocess engineering. Process Biochem. 2017;62:24‐36.

[57] Morrison TM , FDA final guidance. Reporting on computational modeling studies for medical device submissions. [Internet]; 2016 [modified September 21, 2016]. Available from: https://www.fda.gov/downloads/MedicalDevices/DeviceRegulationandGuidance/GuidanceDocuments/Ucomputational_modeling381813.pdf (2016).

[58] Kieninger J , Weltin A , Flamm H , Urban GA . Microsensor systems for cell metabolism ‐ from 2D culture to organ‐on‐chip. Lab Chip. 2018;18:1274‐1291. 29619452 10.1039/c7lc00942a

[59] Clarke GA , Hartse BX , Niaraki Asli AE , et al. Advancement of sensor integrated organ‐on‐chip devices. Sensors). 2021;21. 10.3390/s21041367 PMC7922590 33671996

[60] Castiaux AD , Spence DM , Martin RS . Review of 3D cell culture with analysis in microfluidic systems. Anal Methods. 2019;11:4220‐4232. 32051693 10.1039/c9ay01328h PMC7015157

[61] Mir M , Rivas Torcates L , Zhang YS , Aleman J , Shin SR , Kilic T , Kim D , Mousavi Shaegh SA , Massa S , Riahi R , Chae S , Hu N , Avci H , Zhang W , Silvestri A , Sanati Nezhad A , Manbohi A , De Ferrari F , Polini A , Calzone G , Shaikh N , Alerasool P , Budina E , Kang J , Bhise N , Ribas J , Pourmand A , Skardal A , Shupe T , Bishop CE , Dokmeci MR , Atala A , Khademhosseini A. Multisensor‐integrated organs‐on‐chips platform for automated and continual in situ monitoring of organoid behaviors. Proc Nat Acad Sci. 2017;114:E2293–E2302. 28265064 10.1073/pnas.1612906114 PMC5373350

[62] Kong D , Lv Z , Häring M , et al. In vivo optochemical control of cell contractility at single‐cell resolution. EMBO Rep. 2019;20:e47755. 31663248 10.15252/embr.201947755 PMC6893293

[63] Saffioti NA , Cavalcanti‐Adam EA , Pallarola D . Biosensors for studies on adhesion‐mediated cellular responses to their microenvironment. Front Bioeng Biotechnol. 2020;8:597950. 33262979 10.3389/fbioe.2020.597950 PMC7685988

[64] Aleman J , Kilic T , Mille LS , et al. Microfluidic integration of regeneratable electrochemical affinity‐based biosensors for continual monitoring of organ‐on‐a‐chip devices. Nat Protoc. 2021;16:2564‐2593. 33911259 10.1038/s41596-021-00511-7

[65] Fuchs S , Johansson S , Tjell AØ , et al. In‐line analysis of organ‐on‐chip systems with sensors: integration, fabrication, challenges, and potential. ACS Biomater Sci Eng. 2021;7:2926‐2948. 34133114 10.1021/acsbiomaterials.0c01110 PMC8278381

[66] Picollet‐D'hahan N , Zuchowska A , Lemeunier I , Le Gac S . Multiorgan‐on‐a‐chip: a systemic approach to model and decipher inter‐organ communication. Trends Biotechnol. 2021;39:788‐810. 33541718 10.1016/j.tibtech.2020.11.014

[67] Shirsat NP , English NJ , Glennon B , Al‐Rubeai M . Modelling of mammalian cell cultures. In: Al‐Rubeai M , ed. *Animal cell culture*. *Cell Engineering* . Springer; 2015:259‐326.

[68] Kwist K , Bridges WC , Burg KJL . The effect of cell passage number on osteogenic and adipogenic characteristics of D1 cells. Cytotechnology. 2016;68:1661‐1667. 26208915 10.1007/s10616-015-9883-8 PMC4960171

[69] Lo Surdo J , Bauer SR . Quantitative approaches to detect donor and passage differences in adipogenic potential and clonogenicity in human bone marrow‐derived mesenchymal stem cells. Tissue Eng Part C Methods. 2012;18:877‐889. 22563812 10.1089/ten.tec.2011.0736 PMC3483050

[70] Lossdorfer S , Fiekens D , Salik MI , et al. Subculture affects the phenotypic expression of human periodontal ligament cells and their response to fibroblast growth factor‐2 and bone morphogenetic protein‐7 in vitro. J Periodontal Res. 2008. 10.1111/j.1600-0765.2008.01087.x 18624939

[71] Wall ME , Bernacki SH , Loboa EG . Effects of serial passaging on the adipogenic and osteogenic differentiation potential of adipose‐derived human mesenchymal stem cells. Tissue Eng. 2007;13:1291‐1298. 17518709 10.1089/ten.2006.0275

[72] Lewis MC , Macarthur BD , Malda J , et al. Heterogeneous proliferation within engineered cartilaginous tissue: the role of oxygen tension. Biotechnol Bioeng. 2005;91:607‐615. 16025534 10.1002/bit.20508

[73] Provin C , Takano K , Yoshida T , et al. Low O2 metabolism of HepG2 cells cultured at high density in a 3D microstructured scaffold. Biomed Microdevices. 2009;11:485‐494. 19082898 10.1007/s10544-008-9254-8

[74] Weise F , Fernekorn U , Hampl J , et al. Analysis and comparison of oxygen consumption of HepG2 cells in a monolayer and three‐dimensional high density cell culture by use of a matrigrid®. Biotechnol Bioeng. 2013;110:2504‐2512. 23568058 10.1002/bit.24912

[75] Weltin A , Hammer S , Noor F , et al. Accessing 3D microtissue metabolism: lactate and oxygen monitoring in hepatocyte spheroids. Biosens Bioelectron. 2017;87:941‐948. 27665516 10.1016/j.bios.2016.07.094

[76] Brennan MD , Rexius‐Hall ML , Elgass LJ , Eddington DT . Oxygen control with microfluidics. Lab Chip. 2014;14:4305‐4318. 25251498 10.1039/c4lc00853g

[77] Mehta G , Mehta K , Sud D , et al. Quantitative measurement and control of oxygen levels in microfluidic poly(dimethylsiloxane) bioreactors during cell culture. Biomed Microdevices. 2007;9:123‐134. 17160707 10.1007/s10544-006-9005-7

[78] Abaci HE , Devendra R , Smith Q , et al. Design and development of microbioreactors for long‐term cell culture in controlled oxygen microenvironments. Biomed Microdevices. 2012;14:145‐152. 21947550 10.1007/s10544-011-9592-9

[79] Ochs CJ , Kasuya J , Pavesi A , Kamm RD . Oxygen levels in thermoplastic microfluidic devices during cell culture. Lab Chip. 2014;14:459‐462. 24302467 10.1039/c3lc51160j PMC4305448

[80] Haselgrove JC , Shapiro IM , Silverton SF . Computer modeling of the oxygen supply and demand of cells of the avian growth cartilage. Am J Physiol. 1993;265:C497‐C506. 8368277 10.1152/ajpcell.1993.265.2.C497

[81] Zhang J , Nuebel E , Wisidagama DRR , et al. Measuring energy metabolism in cultured cells, including human pluripotent stem cells and differentiated cells. Nat Protoc. 2012;7:1068‐1085. 22576106 10.1038/nprot.2012.048 PMC3819135

[82] Azarin SM , Larson EA , Almodóvar‐Cruz JM , et al. Effects of 3D microwell culture on growth kinetics and metabolism of human embryonic stem cells. Biotechnol Appl Biochem. 2012;59:88‐96. 23586789 10.1002/bab.1003 PMC3628806

[83] Higuera G , Schop D , Janssen F , et al. Quantifying in vitro growth and metabolism kinetics of human mesenchymal stem cells using a mathematical model. Tissue Eng Part A. 2009;15:2653‐2663. 19207045 10.1089/ten.TEA.2008.0328

[84] Prill S , Jaeger MS , Duschl C . Long‐term microfluidic glucose and lactate monitoring in hepatic cell culture. Biomicrofluidics. 2014;8:34102. 10.1063/1.4876639 PMC4032397 24926387

[85] Devarapalli M , Lawrence BJ , Madihally SV . Modeling nutrient consumptions in large flow‐through bioreactors for tissue engineering. Biotechnol Bioeng. 2009;103:1003‐1015. 19422037 10.1002/bit.22333 PMC2730170

[86] Magrofuoco E , Elvassore N , Doyle FJ . Theoretical analysis of insulin‐dependent glucose uptake heterogeneity in 3D bioreactor cell culture. Biotechnol Progr. 2012;28:833‐845. 10.1002/btpr.1539 22488757

[87] Ho S‐Y , Yu M‐H , Chung CA , Simulation of cell growth and diffusion in tissue engineering scaffolds. In: Magjarevic R , Nagel JH , Lim CT , Goh JCH, eds. 13th International Conference on Biomedical Engineering: ICBME 2008, 3–6 December 2008, Singapore. IFMBE Proceedings, 23, v.v. 23 (1st ed.). Springer‐Verlag; 2009:1745‐1748.

[88] Galban CJ , Locke BR . Analysis of cell growth kinetics and substrate diffusion in a polymer scaffold. Biotechnol Bioeng. 1999;65:121‐132. 10458732 10.1002/(sici)1097-0290(19991020)65:2<121::aid-bit1>3.0.co;2-6

[89] Chung CA , Chen CW , Chen CP , Tseng CS . Enhancement of cell growth in tissue‐engineering constructs under direct perfusion: modeling and simulation. pdf. Biotechnol Bioeng. 2007;97:1603‐1616. 17304558 10.1002/bit.21378

[90] Dunn JC . Analyses of cell growth in tissue‐engineering scaffolds. Regen Med. 2008;3:421‐424. 18462062 10.2217/17460751.3.3.421

[91] Braun & Beatty Microvas Res 2007.

[92] Busek M , Grünzner S , Steege T , et al. Hypoxia‐on‐a‐chip. Curr Dir Biomed. 2016;2:71‐75.

[93] Eibl R , Catapano G , Czermak P , et al. *Cell and Tissue Reaction Engineering: With a Contribution by Martin Fussenegger and Wilfried Weber*. *Principles and Practice* . Springer, Berlin, Heidelberg; 2009.

[94] Guarino RD , Dike LE , Haq TA , et al. Method for determining oxygen consumption rates of static cultures from microplate measurements of pericellular dissolved oxygen concentration. Biotechnol Bioeng. 2004;86:775‐787. 15162453 10.1002/bit.20072

[95] Deshpande RR , Heinzle E . On‐line oxygen uptake rate and culture viability measurement of animal cell culture using microplates with integrated oxygen sensors. Biotechnol Lett. 2004:26. 10.1023/b:bile.0000024101.57683.6d 15195979

[96] Oomen PE , Skolimowski MD , Verpoorte E . Implementing oxygen control in chip‐based cell and tissue culture systems. Lab Chip. 2016;16:3394‐3414. 27492338 10.1039/c6lc00772d

[97] Lin Z , Cherng‐Wen T , Roy P , Trau D . In‐situ measurement of cellular microenvironments in a microfluidic device. Lab Chip. 2009;9:257‐262. 19107282 10.1039/b806907g

[98] Ungerböck B , Charwat V , Ertl P , Mayr T . Microfluidic oxygen imaging using integrated optical sensor layers and a color camera. Lab Chip. 2013;13:1593‐1601. 23443957 10.1039/c3lc41315b

[99] Jomezadeh Kheibary N , Abolfazli Esfahani J , Mousavi Shaegh SA . Analysis of oxygen transport in microfluidic bioreactors for cell culture and organ‐on‐chip applications. Struct Eng Rep. 2020;2.

[100] Christensen MG , Cawthorne C , Dyer CE , et al. Investigating oxygen transport efficiencies in precision‐cut liver slice‐based organ‐on‐a‐chip devices. Microfluid Nanofluid. 2021;25.

[101] Mattei G , Giusti S , Ahluwalia A. Design Criteria for Generating Physiologically Relevant In Vitro Models in Bioreactors. Processes. 2014;2:548.–569.

[102] Galbusera F , Cioffi M , Raimondi MT , Pietrabissa R . Computational modeling of combined cell population dynamics and oxygen transport in engineered tissue subject to interstitial perfusion. Comput Meth Biomech Biomed Eng. 2007;10:279‐287. 10.1080/10255840701318404 17671861

[103] Mareels G , Poyck PPC , Eloot S , et al. Three‐dimensional numerical modeling and computational fluid dynamics simulations to analyze and improve oxygen availability in the AMC bioartificial liver. Ann Biomed Eng. 2006;34:1729‐1744. 17031599 10.1007/s10439-006-9169-6 PMC1705524

[104] Cussler EL . *Diffusion: Mass Transfer in Fluid Systems*. *Cambridge Series in Chemical Engineering* (2nd ed.). Cambridge University Press; 2000.

[105] Hsu H‐H , Harder LE , Lindner G , et al. A method to determine and simulate the permeation through a gel matrix in a multi‐organ‐chip. BMC Proc. 2015;9:77.

[106] Pluen A , Netti PA , Jain RK , Berk DA . Diffusion of macromolecules in agarose gels: comparison of linear and globular configurations. Biophys J. 1999;77:542‐552. 10388779 10.1016/S0006-3495(99)76911-0 PMC1300351

[107] Hsu H‐H , Kracht J‐K , Harder LE , et al. A method for determination and simulation of permeability and diffusion in a 3D tissue model in a membrane insert system for multi‐well plates. JOVE‐J VIS EXP. 2018. 10.3791/56412 PMC5931342 29553546

[108] Hadgraft J. Skin, the final frontier. Int J Pharm. 2001;224:1–18. 11512545 10.1016/s0378-5173(01)00731-1

[109] Ng S‐F , Rouse JJ , Sanderson FD , et al. Validation of a static Franz diffusion cell system for in vitro permeation studies. AAPS PharmSciTech. 2010;11:1432‐1441. 20842539 10.1208/s12249-010-9522-9 PMC2974154

[110] Cornelissen LH , Bronneberg D , Oomens CWJ , Baaijens FPT . Diffusion measurements in epidermal tissues with fluorescent recovery after photobleaching. Skin Res Technol. 2008;14:462‐467. 18937782 10.1111/j.1600-0846.2008.00313.x

[111] Leddy HA , Guilak F . Site‐specific molecular diffusion in articular cartilage measured using fluorescence recovery after photobleaching: wichtig.pdf. Ann Biomed Eng. 2003:753‐760. 12971608 10.1114/1.1581879

[112] Schimek K , Hsu H‐H , Boehme M , et al. Bioengineering of a full‐thickness skin equivalent in a 96‐well insert format for substance permeation studies and organ‐on‐a‐chip applications. Bioengineering. 2018;5. 10.3390/bioengineering5020043 PMC6027510 29880746

[113] Ho ST , Hutmacher DW . A comparison of micro CT with other techniques used in the characterization of scaffolds. Biomaterials. 2006;27:1362‐1376. 16174523 10.1016/j.biomaterials.2005.08.035

[114] Bertoldi S , Farè S , Tanzi MC . Assessment of scaffold porosity: the new route of micro‐CT. J Appl Biomater Biomech. 2011;9:165‐175. 22139756 10.5301/JABB.2011.8863

[115] Bohner M , Loosli Y , Baroud G , Lacroix D . Commentary: deciphering the link between architecture and biological response of a bone graft substitute. Acta Biomater. 2011;7:478‐484. 20709195 10.1016/j.actbio.2010.08.008

[116] Dias MR , Fernandes PR , Guedes JM , Hollister SJ . Permeability analysis of scaffolds for bone tissue engineering. J Biomech. 2012;45:938‐944. 22365847 10.1016/j.jbiomech.2012.01.019

[117] Pennella F , Cerino G , Massai D , et al. A survey of methods for the evaluation of tissue engineering scaffold permeability. Ann Biomed Eng. 2013;41:2027‐2041. 23612914 10.1007/s10439-013-0815-5

[118] Schiavi A , Guglielmone C , Pennella F , Morbiducci U . Acoustic method for permeability measurement of tissue‐engineering scaffold. Meas Sci Technol. 2012;23:105702.

[119] Chor MV , Li W . A permeability measurement system for tissue engineering scaffolds. Meas Sci Technol. 2007;18:208‐216.

[120] Shimko DA , Nauman EA . Development and characterization of a porous poly(methyl methacrylate) scaffold with controllable modulus and permeability. J Biomed Mater Res B Appl Biomater. 2007;80:360‐369. 16838352 10.1002/jbm.b.30605

[121] Truscello S , Kerckhofs G , van Bael S , et al. Prediction of permeability of regular scaffolds for skeletal tissue engineering: a combined computational and experimental study. Acta Biomater. 2012;8:1648‐1658. 22210520 10.1016/j.actbio.2011.12.021

[122] Boodt S , Truscello S , Ozcan SE , et al. Bi‐modular flow characterization in tissue engineering scaffolds using computational fluid dynamics and particle imaging velocimetry. Tissue Eng Part C Methods. 2010;16:1553‐1564. 20590471 10.1089/ten.tec.2010.0107

[123] Jones JR , Poologasundarampillai G , Atwood RC , et al. Non‐destructive quantitative 3D analysis for the optimisation of tissue scaffolds. Biomaterials. 2007;28:1404‐1413. 17141863 10.1016/j.biomaterials.2006.11.014

[124] Buerk DG , Saidel GM . Local kinetics of oxygen metabolism in brain and liver tissues. Microvasc Res. 1978;16:391‐405. 748721 10.1016/0026-2862(78)90072-9

[125] Jones DP , Mason HS . Gradients of O2 concentration in hepatocytes. J Biol Chem. 1978;253:4874‐4880. 209020

[126] Xiong Y , Peterson P , Lee C . Polarographic assays of mitochondrial functions. In: Celis JE , ed. Cell Biology: A Laboratory Handbook (3rd ed.). Elsevier; 2006:259‐264.

[127] Rotem A , Toner M , Tompkins RG , Yarmush ML . Oxygen uptake rates in cultured rat hepatocytes. Biotechnol Bioeng. 1992;40:1286‐1291. 18601082 10.1002/bit.260401020

[128] Nyberg SL , Remmel RP , Mann HJ , et al. Primary hepatocytes outperform Hep G2 cells as the source of biotransformation functions in a bioartificial liver. Ann Surg. 1994;220:59‐67. 8024360 PMC1234288

[129] Foy BD , Rotem A , Toner M , et al. A device to measure the oxygen uptake rate of attached cells: importance in bioartificial organ design. Cell Transplant. 1994;3:515‐527. 7881763 10.1177/096368979400300609

[130] Smith MD , Smirthwaite AD , Cairns DE , et al. Techniques for measurement of oxygen consumption rates of hepatocytes during attachment and post‐attachment. Int J Artif Organs. 1996;19:36‐44. 8641817

[131] Balis UJ , Behnia K , Dwarakanath B , et al. Oxygen consumption characteristics of porcine hepatocytes. Metab Eng. 1999;1:49‐62. 10935754 10.1006/mben.1998.0105

[132] Roy P , Baskaran H , Tilles AW , et al. Analysis of oxygen transport to hepatocytes in a flat‐plate microchannel bioreactor. Ann Biomed Eng. 2001;29:947‐955. 11791677 10.1114/1.1415524

[133] Allen JW , Bhatia SN . Formation of steady‐state oxygen gradients in vitro: application to liver zonation. Biotechnol Bioeng. 2003;82:253‐262. 12599251 10.1002/bit.10569

[134] Chow DC , Wenning LA , Miller WM , Papoutsakis ET . Modeling pO2 distributions in the bone marrow hematopoietic compartment. I. Krogh's model. Biophys J. 2001;81:675‐684. 11463616 10.1016/S0006-3495(01)75732-3 PMC1301544

[135] Nehring D , Adamietz P , Meenen NM , Pörtner R . Perfusion cultures and modelling of oxygen uptake with three‐dimensional chondrocyte pellets. Biotechnol Tech. 1999;13:701‐706.

[136] Super A , Jaccard N , Cardoso Marques MP , et al. Real‐time monitoring of specific oxygen uptake rates of embryonic stem cells in a microfluidic cell culture device. Biotechnol J. 2016;11:1179‐1189. 27214658 10.1002/biot.201500479 PMC5103178

